# Emergence of Genomic Diversity in the Spike Protein of the “Omicron” Variant

**DOI:** 10.3390/v15102132

**Published:** 2023-10-21

**Authors:** Surajit Basak, Pratanu Kayet, Manisha Ghosh, Joyeeta Chatterjee, Shanta Dutta

**Affiliations:** 1 Division of Bioinformatics, ICMR-National Institute of Cholera and Enteric Diseases, Kolkata 700010, India; 2 Centre for Bioinformatics, School of Life Sciences, Pondicherry University, Pondicherry 605014, India; 3 Division of Bacteriology, ICMR-National Institute of Cholera and Enteric Diseases, Kolkata 700010, India

**Keywords:** SARS-CoV-2, VOC, amino acid usage, mutation rate, recombination, receptor binding domain

## Abstract

SARS-CoV-2 (Severe Acute Respiratory Syndrome Coronavirus) has constantly been evolving into different forms throughout its spread in the population. Emerging SARS-CoV-2 variants, predominantly the variants of concern (VOCs), could have an impact on the virus spread, pathogenicity, and diagnosis. The recently emerged “Omicron” variant has exhibited rapid transmission and divergence. The spike protein of SARS-CoV-2 has consistently been appearing as the mutational hotspot of all these VOCs. In order to determine a deeper understanding of the recently emerged and extremely divergent “Omicron”, a study of amino acid usage patterns and their substitution patterns was performed and compared with those of the other four successful variants of concern (“Alpha”, “Beta”, “Gamma”, and “Delta”). We observed that the amino acid usage of “Omicron” has a distinct pattern that distinguishes it from other VOCs and is significantly correlated with the increased hydrophobicity in spike proteins. We observed an increase in the non-synonymous substitution rate compared with the other four VOCs. Considering the phylogenetic relationship, we hypothesized about the functional interdependence between recombination and the mutation rate that might have resulted in a shift in the optimum of the mutation rate for the evolution of the “Omicron” variant. The results suggest that for improved disease prevention and control, more attention should be given to the significant genetic differentiation and diversity of newly emerging variants.

## 1. Introduction

Severe acute respiratory syndrome coronavirus 2 (SARS-CoV-2) responsible for the Coronavirus disease 2019 (COVID-19) pandemic has had a distressing effect globally [1,2]. The number of mutations that accumulate as the genome replicates increases, leading to the variants being produced. The emergence of SARS-CoV-2 has also generated interest in the role of recombination in the evolution of the virus [3].

Distinguished SARS-CoV-2 variants were categorized by the World Health Organization (WHO) into three groups: variants of concern (VOCs), variants of interest (VOIs), and variants under monitoring (VUMs) in order to prioritize the surveillance of these variants [4,5]. Evolution of mutated lineages with higher infectivity or immune escape capacities were eventually categorized as VOCs based on their characteristics [4]. As of December 2021, WHO has identified five VOCs (“Alpha”, “Beta”, “Gamma”, “Delta”, and “Omicron”). Among them, the “Alpha” VOC (Pango lineage B.1.1.7) was first detected in the U.K. in 2020, the “Beta” VOC (Pango lineage B.1.351) was first detected in South Africa in 2020, the “Gamma” VOC (Pango lineage P.1) was first detected in 2020 in Brazil, the “Delta” VOC (Pango lineage B.1.617.2) was first detected in India in late 2020, and the “Omicron” VOC (Pango lineage B.1.1.529) was first detected in South Africa and Botswana in November 2021. The “Omicron” variant outcompeted other variants with a greater number of mutations, specifically in the spike (S) protein, which has been linked to the high transmissibility and infectivity of this variant [6,7,8].

The evolution and adaptability of the viral genome to the host genome have been related to the global spread of SARS-CoV-2, which has shown significant drops in some places and sharp increases in others in terms of transmission rates. The variability has also been altered by variations in host immune systems, mutations, deletions, recombination, and genetic drift [9]. Studies have shown that the patterns of global variation are probably caused by adaptations at the nucleotide and amino acid regions as well as the variability found in structural proteins, notably spike proteins [10]. Multivariate analysis of the amino acid usage pattern will help to understand the worldwide heterogeneity of SARS-CoV-2, the evolution of its genome, and its adaptability to the host. Studies have also shown natural selection and mutational pressure as key factors influencing the variation in SARS-CoV-2 [3,11].

Genome-wide analysis of SARS-CoV-2 variants with the first emerged genome in Wuhan city in China in 2019 might suggest a relative association among viral strains [9,12]. With the prior knowledge of the accumulation of nucleotide substitution over time, the estimation of the most recent common ancestor (tMRCA) of the two strains is possible [13]. As the SARS-CoV-2 genome is almost entirely made up of protein-coding regions, it is crucial to distinguish between nonsynonymous and synonymous substitution rates [14,15]. The SARS-CoV-2 genome can actually be thought of as a collection of several “recombination blocks”, or areas between predicted breakpoints for recombination events [16]. For the current pandemic, recombination events in the evolutionary history of the spike protein are specifically important [17].

This study aims to comprehend the evolutionary pattern and mutational landscape of the five VOCs (“Alpha”, “Beta”, “Delta”, “Gamma”, and “Omicron”). First, we will identify the major trends in amino acid usage of spike proteins across five VOCs through multivariate analysis. We will also evaluate the evolutionary perspective, such as nonsynonymous and synonymous substitutions rates and rate of evolution, and then analyze the recombination pattern of the five VOCs. This study will be important to understand the mutational and evolutionary properties that are necessary for new therapeutic and vaccine development to combat the virus.

## 2. Materials and Methods

### 2.1. Sequence Retrieval

A total of 456,409 spike nucleotide sequences of SARS-CoV-2 were downloaded from the NCBI-Virus repository (https://www.ncbi.nlm.nih.gov/sars-cov-2 accessed on 20 March 2022). Partial sequences and sequences containing ambiguous characters were excluded from our dataset [1,18]. NCBI provides information about each spike sequence regarding its association with a designated VOC (i.e., “Alpha”/“Beta”/“Gamma”/“Delta”/“Omicron”). Finally, there were 100,309, 427, 175,351, 9618, and 170,243 numbers of “Alpha”, “Beta”, “Delta”, “Gamma”, and “Omicron” variant sequences, respectively, in the downloaded data (Supplementary Table S1). 250 whole genome sequences covering five VOCs of SARS-CoV-2 were downloaded from NCBI for the recombination study. Angiotensin-converting enzyme 2 (ACE2) variants were retrieved from the Genome Aggregation Consortium Database (gnomAD) (https://gnomad.broadinstitute.org/ accessed on 1 September 2023) (Supplementary Table S2).

### 2.2. Correspondence Analysis on Amino Acid Usage

To assess the variations in the amino acid usage of the spike protein, we performed a correspondence analysis (CoA). Major trends in variance in the dataset were revealed by placing the data along continuous axes. We employed correspondence analysis available in CodonW v1.4.2 software for the amino acid usage analysis of spike gene sequences [19,20,21]. Determination of the hydrophobicity of each spike gene sequence was conducted using the Kyte–Doolittle method present in the CodonW program [1,22].

### 2.3. Analysis of Evolutionary Selection

The ratio (ω) of the rate of non-synonymous substitutions per nonsynonymous site (Ka) to the rate of synonymous substitutions per synonymous site (Ks) reveals the influence of evolution on a gene segment. ω > 1 indicates diversifying (positive) selection, whereas ω < 1 signifies purifying (negative) selection. The evolutionary rates of genes (with reference to consensus sequence) were calculated using the Codeml program included in the PAML software package (ver. 4.5) [23,24,25,26] (http://abacus.gene.ucl.ac.uk/software/paml.html accessed on 1 September 2023) with runmode = −2 and CodonFreq = 1 [26]. Spike gene sequences were subjected to the analysis of synonymous and non-synonymous substitution rates with respect to the reference SARS-CoV-2 spike gene sequence. Statistical tests such as the t-test were conducted using the GraphPad (https://www.graphpad.com/ accessed on 1 September 2023) web application.

### 2.4. Detection of Mutation Rate and TMRCA

BEAST (Bayesian Evolutionary Analysis Sampling Trees) is a software suite for phylogenetic analysis with an importance on time-scaled trees. The BEAST-1.10.4 software package was used to estimate the mutation rate and TMRCA (Time to Most Recent Common Ancestor) of the spike gene sequences [27]. Gene sequences were aligned using MAFFT v.7 [28] and the best-fit evolutionary model was estimated in MEGA-X [29]. The BEAUti2 v1.10.4 graphical user interface tool was used for generating the configuration files. The Tracer [30] and FigTree v1.4.4 tools were used for analyzing and visualizing the log data.

### 2.5. Recombination Analysis

A recombination analysis was performed with the whole genome sequences using the RDP4 program [31]. From the GISAID database, we have taken one whole genome from each lineage of all variants of concern (VOCs) of SARS-CoV-2 for our analysis and aligned them against the reference SARS-CoV-2 genome using MAFFTv7 [28]. The full-genome alignment was scanned for recombination using different algorithms. RDP, GENECONV, MaxChi, Chimera, and 3Seq algorithms were used for the primary scan. BootScan and SiScan algorithms were used for the secondary scan [32,33,34].

### 2.6. Protein Homology Modeling and Docking

Three-dimensional structural models of the spike protein of five VOCs were generated through homology modeling using the MODELER program [35]. Similarly, the structure of angiotensin-converting enzyme 2 (ACE2) was also generated. Protein structural models generated through homology modeling were refined using the ModRefiner web server [36]. The molecular interaction between viral spike proteins and the human ACE2 receptor was studied using a Z-dock server [37]. Then, using the PRODIGY webserver [38], the resulting docking data were processed and analyzed while taking the binding energies of each complex into consideration.

## 3. Results

### 3.1. Analysis of Amino Acid Usage

**Figure 1:**
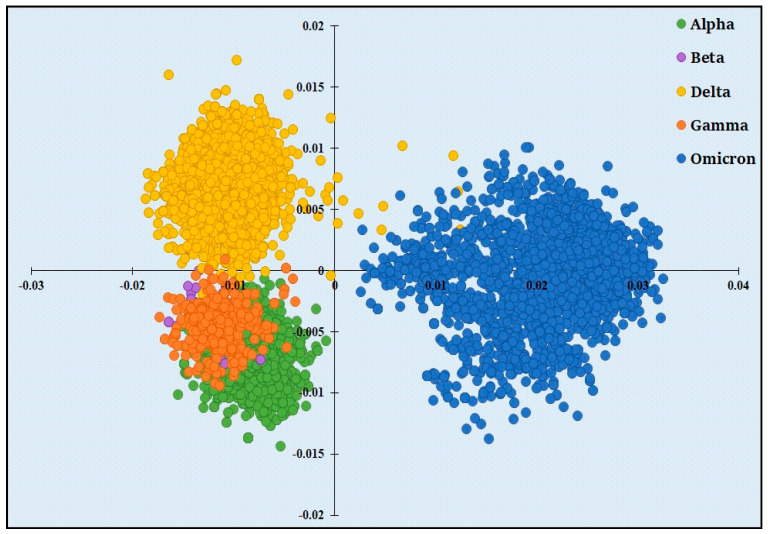
Distribution of spike genes of five VOCs along the two major axes of the correspondence analysis (COA) based on amino acid usage (AAU) data. *x*-axis−Axis 1 of AAU; *y*-axis—Axis 2 of AAU. Spike genes of “Alpha”, “Beta”, “Delta”, “Gamma”, and “Omicron” are represented with green, purple, yellow, orange, and blue dots, respectively.

We performed a correspondence analysis to detect the amino acid usage pattern among the spike gene sequences of five SARS-CoV-2 variants. We observed a clear separation of the spike genes of the “Omicron” variant from the other four variants along the first major axis (Figure 1) that explained 82.86% of the total variations, while no other axis could explain more than 9.36% of the total variations.

We observed a positive correlation between the hydrophobicity of the encoded proteins and the position of the genes along the horizontal axis (r = 0.238, *p* < 0.01). Additionally, we found that the average hydrophobicity of the spike proteins distributed on the negative side (−0.0821) of the horizontal axis is significantly lower (*p* < 0.01) than the average hydrophobicity of the spike proteins distributed on the positive side (−0.0795) of the horizontal axis. These results clearly state that the evolution of hydrophobicity in spike proteins has been associated with the accumulation of more hydrophobic residues in the “Omicron” variant with respect to “Delta” and “Alpha” variants.

According to the amino acid composition, there is a rise in the following amino acid compositions of the “Omicron” variant compared with the “Delta” variant: Arginine, Lysine, Aspartic acid, and Glutamic acid. These increases indicate that the “Omicron” variant has more charged residues that contribute to salt bridge formation and that charged residues are exposed to a much greater degree.

The higher amino acid composition of Phenylalanine and Isoleucine in the “Omicron” spike protein, when compared with the “Delta” variant, suggests that the “Omicron” spike protein includes more hydrophobic amino acids, which may be due to its positioning inside the protein core. When compared with the “Delta” variant, the “Omicron” variant’s amino acid composition is low in polar amino acids such as Asparagine and Glutamine.

### 3.2. Evolutionary Rate Analysis

Estimation of synonymous (Ks) and nonsynonymous (Ka) substitution rates is important in understanding the dynamics of molecular sequence evolution. The Ka values for the spike gene sequences were found to correlate significantly with the data points on Axis 1 (r = 0.98, *p* < 0.01). The Ks values also correlated significantly with the data points on Axis 1 (r = 0.91, *p* < 0.01). These results indicate that evolutionary selection pressure significantly influenced the amino acid usage pattern of spike gene sequences of five variants of SARS-CoV-2.

**Figure 2:**
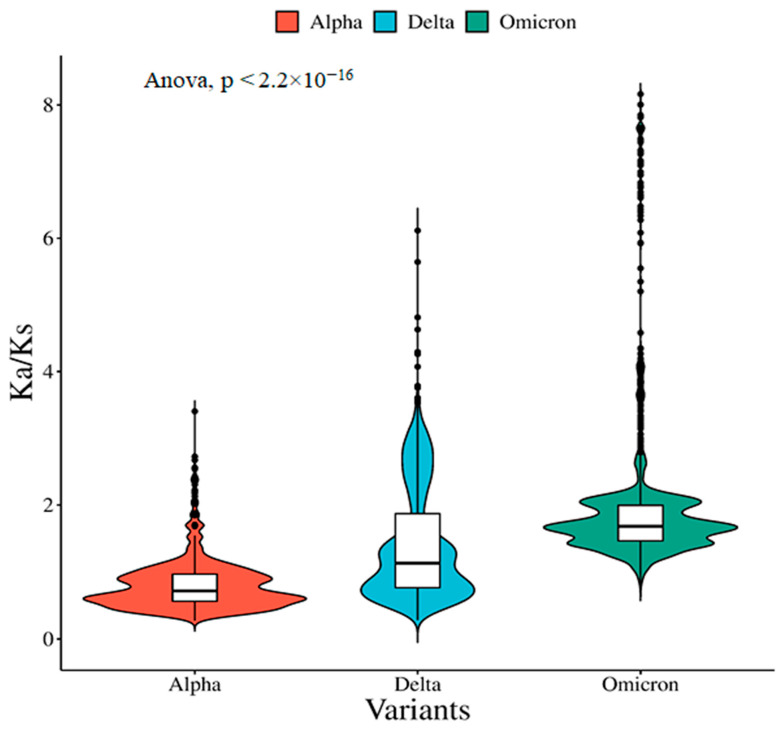
Distribution and statistical comparison of the Ka/Ks ratio of spike genes among the “Alpha”, “Delta”, and “Omicron” variants. The plot shows a significantly higher distribution of the Ka/Ks ratio of the “Omicron” variant compared with the “Alpha” and “Delta” variants.

Our results show that the Ka/Ks values of the spike genes of the “Alpha” variant are the lowest and under purifying selection. Gradually, the values of Ka/Ks increase to reach the value closer to 1 (1.008) for the “Delta” variant, indicating that the genome was going through a neutral evolution (Figure 2). The values of Ka/Ks become much higher for the “Omicron” variant and under positive selection. This increase in Ka/Ks is mostly due to the enhanced rate of the nonsynonymous substitution rate; in particular, the nonsynonymous substitution rate is increased more than four times in the “Omicron” variant compared with the “Delta” variant. We hypothesize that this increase may be attributed to a larger diversity of sequences that may have given rise to more diverse lineages via undetected intra-SARS-CoV-2 recombination, which is analogous to a positive feedback loop.

The relatively high Ka/Ks (Figure 2) ratio for the “Omicron” variant suggests that the selective pressure acting on the spike protein of the “Omicron” variant is relaxed, and some sites may be undergoing positive selection. This increased evolutionary rate can be explained by the important function of the spike protein, which participates in host-specific recognition and undergoes several drastic changes during virus infection.

### 3.3. Interaction Profile between Spike Protein and ACE2

**Figure 3:**
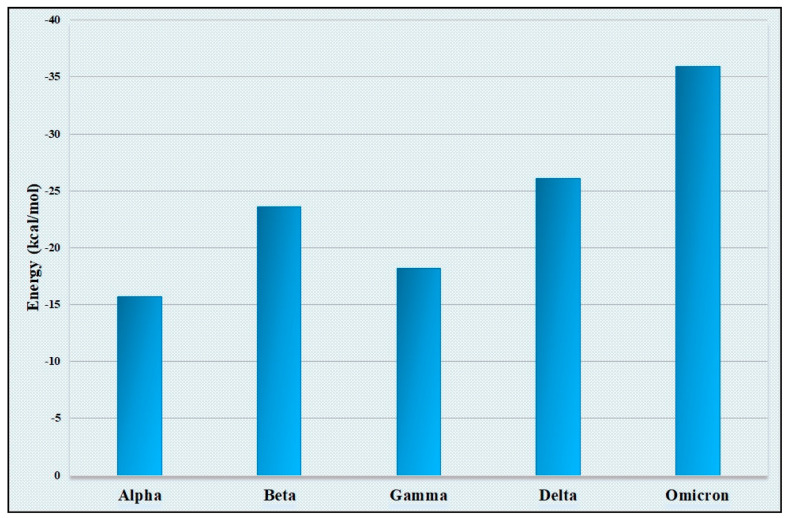
Variation in the binding energy of the spike−ACE2 complex among the five VOCs showing lowest binding energy (energy in negative scale) in the “Omicron” variant.

Our comparison of amino acid usage underlines the differential pattern of evolution through the accumulation of mutations in spike proteins among the five SARS-CoV-2 variants. Since the receptor for SARS-CoV-2 has been identified as ACE2, it was very important to analyze how the differential mutation patterns of amino acid usages of the spike proteins of five variants of SARS-CoV-2 responded to binding to the human ACE2 receptor. Three-dimensional structures of spike protein sequences for spike proteins from each of the five variants were constructed through homology modeling. The 3D structure of ACE2 was also generated computationally through homology modeling. The docking study was performed with ACE2 separately with five spike proteins representing five different VOCs and the binding energy was calculated separately for each of the docking experiments. We observed that the binding energy for the spike–ACE2 complex for the “Omicron” variant is lowest among the binding energy of the four other complexes (Figure 3). The “Omicron” variant always represented the lowest energy complex with all the ACE2 variants when the ACE2 variants were docked with the spike protein variants. A lower binding energy for the spike–ACE2 complex for the “Omicron” variant indicates its higher stability compared with the other four complexes made by the ACE2 and spike protein from the “Alpha”, “Beta”, “Gamma” and “Delta” variants. The highest stability of the spike (“Omicron”)-ACE2 complex may also be corroborated by the presence of more hydrogen bonds in the spike (“Omicron”)-ACE2 complex (Supplementary Figure S1).

We surveyed the Genome Aggregation Consortium Database (gnomAD) (https://gnomad.broadinstitute.org/ accessed on 1 September 2023) and found that human ACE2 is highly polymorphic, with single-nucleotide variants (SNVs) that result in missense mutations. These ACE2 could influence susceptibility to SARS-CoV-2 and potentially affect disease outcomes. The information on the association of each of these ACE2 alleles in various populations (e.g., East Asian, South Asian, European, African, Admixed American, etc.) was collected from the same database. The number of ACE2 genes associated with each population is shown in Table 1. For a given population, the allele frequency was calculated and the allele with the highest frequency is considered as the most common allele in the given population. The binding energies between the most common allele of a population and the spike protein of Omicron are provided in Table 2. It is clear that the highest binding energy is represented by four different populations (viz. American, Jewish, European, and South Asian). African and East Asian populations represent lower binding energies. Our results support epidemiological evidence that the African continent has an extremely low incidence and fatality rate compared with America [39]. We observed lower binding energy for the African population compared with the American population, which, in turn, indicates that the African population will be less prone to SARS-CoV-2 infection due to enhanced binding affinity.

### 3.4. Mutation Rate and tMRCA

In this study, tMRCA results were in accordance with the reported emergence of all VOCs (designated as per WHO guidelines). The “Beta” and “Gamma” variants appeared almost simultaneously, followed by the “Delta” variant and then the “Omicron” variant (Table 3). This observation in time scale phylogeny helps us to understand that, in most cases, recombinant “Delta” lineages were created by other “Delta” linages because all the “Delta” lineages shared a common ancestor at a time point during the 2nd wave of COVID-19.

**Figure 4:**
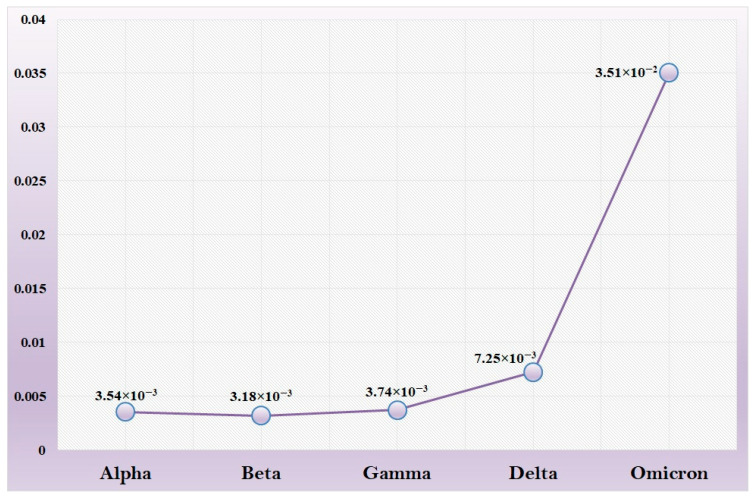
Graphical representation of the mutation rate of five VOCs. A ten−time increase in mutation rate is observed in the “Omicron” variant compared with the “Alpha” variant.

Recombination events in the evolutionary history of the spike protein have particular significance for the current pandemic. The spike protein sequences are known to undergo frequent changes through recombination. The recombination and tMRCA results show us that some of the early recombinant lineages of a particular variant were created by intra-variant recombination, e.g., recombination between several lineages of “Delta” like AY.80, AY.86, etc. But later recombinants like BA.2 were the results of inter-variant recombination (Table 4). In the case of the “Omicron” variant, we observed a nearly 10-time increase in mutation rate compared with the “Alpha” variant (Figure 4). The enhanced mutation rate might have been possible due to the higher rate of nonsynonymous substitution as observed in this study. The “Delta” and “Omicron” variants created recombinants like BA.2 because they all share a common ancestor in time scale phylogeny.

## 4. Discussion

This study compared the amino acid usage patterns of spike proteins of five VOC lineages (“Alpha”, “Beta”, “Gamma”, “Delta”, and “Omicron”). The distinct amino acid usage pattern of the “Omicron” variant from other variants is clear in Figure 1. The selection pressure on the evolution of spike genes in “Omicron” is expected to affect the distinct amino acid usage pattern. The non-synonymous (Ka) to synonymous substitutions (Ks) ratio in protein-coding genes is commonly used to detect the selection pressure during gene evolution. A Ka/Ks ratio larger than 1 indicates positive selection, while a Ka/Ks ratio less than 1 indicates negative selection acting on protein-coding genes. Our results show that the Ka/Ks of the “Alpha” variant of SARS-CoV-2 is less than 1 indicating purifying selection. The “Delta” variant showed a value almost equal to 1 indicating neutral selection. However, the comparatively high Ka/Ks ratio of the spike protein of the “Omicron” variant indicates that the selective pressure acting on this variant is relaxed, and positive selection may be occurring in some sites. This accelerated evolutionary rate can be explained by the crucial role of the spike protein, which contributes to host-specific recognition and goes through a number of significant modifications during virus infection.

Furthermore, our results from mutation rate analysis suggest that the rate of mutation significantly increases from the “Delta” to the “Omicron” variant. Observations from evolutionary selection suggest that “Alpha” variants face relatively greater purifying selection pressure during the 1st wave of the pandemic. But this scenario was changed during the 2nd wave, which was caused by the “Delta” variant. The “Delta” variant and its lineages appear to have been subjected to nearly neural selection pressure from the beginning to the end of the wave. Again, these scenarios were changed during the emergence of the “Omicron” variant, which is associated with positive selection pressure.

Thus, it is worth noting that the rate of substitution is an important driving force for evolutionary selection, and the variation in evolutionary selection pressure may be critical for various waves throughout the pandemic period. Purifying selection pressure is associated with genomes of the “Alpha” variant during the first wave of the pandemic whose duration was shorter than the other two waves. In the case of the “Omicron” variant, positive evolutionary selection helps to establish its lineages in the human population longer than the other two variants through stronger binding affinity with the ACE2 receptor.

**Figure 5:**
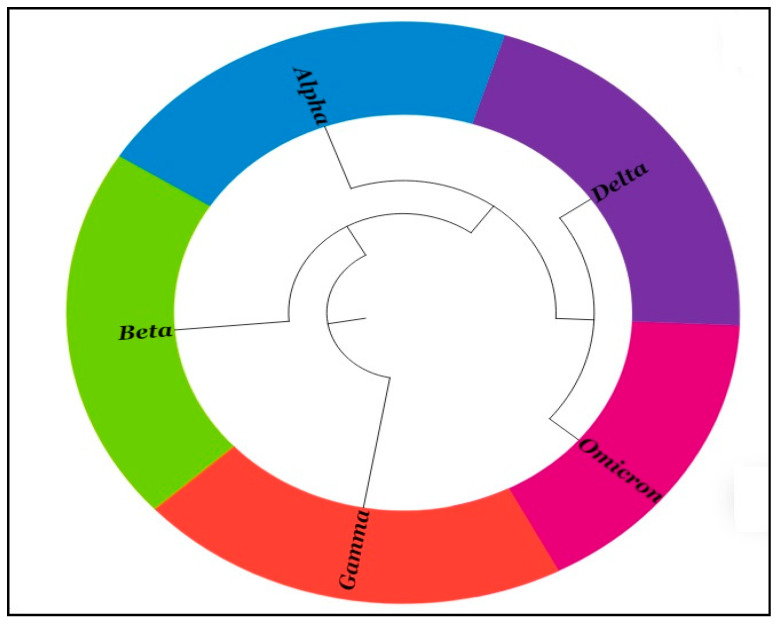
The phylogenetic tree among the variants shows that one group consists of “Alpha”, “Beta”, “Delta”, and “Omicron”, and another group consists of “Gamma”.

The phylogenetic tree (Figure 5) among the variants displays two clades formed after the divergence from the root. One clade includes “Alpha” (blue), “Beta” (green), “Delta” (violet), and “Omicron” (pink), and the other clade includes “Gamma” (red).

Our data show that the number of recombinants started increasing from the “Delta” variant, but its mutation rate was similar to other variants like “Alpha”, “Beta”, and “Gamma”. After that, a number of recombinants and a nearly 10-time increase in the mutation rate of the “Omicron” variant was observed. As an explanation for this occurrence, we prepared two hypotheses: one for the “Delta” variant, where we observed no shift in the mutation rate despite recombination. The other one is for the “Omicron” variant, where we observed a shift in mutation rate along with the recombination.

Hypothesis I: Recombination could accelerate adaptation without changing the optimal mutation rate. Earlier in the pandemic, diversity among SARS-CoV-2 was low and, eventually, the recombination rate was low. But later in the pandemic, various “Delta” lineages were created as a result of recombination. After this happened, recombination could speed up adaptation without changing the optimal mutation rate. This led to more differences between “Delta” lineages, but it did not change the optimal mutation rate.

Hypothesis II: “Omicron” was a product of the recombination of “Omicron” and “Delta” lineages. As a result, by the time of the emergence of this variant, genomic variation among the SARS-CoV-2 genomes was very high. Where there is a functional link between recombination and mutation, the rate of mutation shifts, and the ability of the host to adapt increases. Functional interdependence between recombination and the mutation rate would result in a shift in the optimum of the mutation rate.

The increase in the mutation rate in “Omicron” subvariants compared with other variants led to an increase in infectivity [40,41]. Although the accumulation of effective mutations is generally considered to be the main driving force behind viral evolution [42], inter-variant and intra-variant recombination can actually escalate this evolution process [43]. The high genetic diversity among the SARS-CoV-2 genomes during the time of evolution of the “Omicron” variant might be an important reason behind the large diversity observed among “Omicron” subvariants. The co-occurrence of multiple subvariants at a similar time has made it possible for “Omicron” to undergo multiple recombinations due to co-infection of the host by different variants [44].

The increased recombination event in the “Omicron” variant has led to changes in the binding affinity between the Receptor Binding Domain (RBD) region of the spike protein and the human receptor ACE2. The increase in recombination rate will result in an increase in mutation rate in order to better the adaption capability of the virus. The high mutation rate in the “Omicron” variant increased the probability of accumulation of effective mutation in the spike proteins which serve as the key region in the viral genome to boost viral adaptation to the host. “Omicron” subvariants have a much higher rate of effective mutation among the other SARS-CoV-2 variants, which characterizes the viral adaptability through better binding of spike proteins to human receptors, thus increasing its infectivity.

## 5. Conclusions

The process of viral evolution is persistent and has the potential to enhance “viral fitness” and selective adaption. Scientists and authorities all around the world have suffered as a result of newly emerging SARS-CoV-2 strains. Vaccines presently offer good protection against all VOCs, but continuous monitoring of vaccine effectiveness is necessary to combat the main SARS-CoV-2 strains and the newly emerging variants. The advent of the “Omicron” variant and the evolution of the entire coronavirus subfamily serve as a warning to researchers, scientists, vaccine developers, and policymakers to maintain vigilance. However, the majority of currently approved vaccination plans and monoclonal antibody treatments target the spike ORFs because of their inherently higher rate of mutation and recombination; however, other more stable genomic regions should be thoroughly explored as potential targets for future research.

## Figures and Tables

**Table 1:** The number of ACE2 genes associated with each population.

Population	The Number of Associated ACE2 Genes
African/African American	45
Latino/Admixed American	41
Ashkenazi Jewish	3
East Asian	26
Europe	139
South Asian	43

**Table 2:** The binding energies between the most common allele of a population and spike protein of Omicron.

Population	Most Common Allele	Binding Energy (kcal/mol)
African/African American	rs147311723	−23.4
Latino/Admixed American	rs4646116	−24.2
Ashkenazi Jewish	rs41303171	−24.2
East Asian	rs191860450	−23.9
Europe	rs41303171	−24.2
South Asian	rs41303171	−24.2

**Table 3:** TMRCA values depicting the emergence of the VOCs. A ten-time increase in mutation rate is observed in the “Omicron” variant compared with the “Alpha” variant.

Spike Variant	Mutation Rate	Mean Value (TMRCA)	Root Age
“Alpha”	3.537 × 10^−3^	1.109	2019.943
“Beta”	3.18 × 10^−3^	0.95	2020.03
“Gamma”	3.737 × 10^−3^	1.07	2020.006
“Delta”	7.25 × 10^−3^	1.189	2020.967
“Omicron”	3.506 × 10^−2^	0.746	2021.414

**Table 4:** Recombination analysis demonstrated that several recombinant events occurred among variants highlighted in the table. All recombinants and their respective major and minor parents are listed below. All are significant at *p* < 0.01.

Recombinant	Major Parent	Minor Parent
“Delta” AY99.1	“Delta” AY.126	“Delta” AY.106
“Delta” AY.88	“Gamma” P.1.5	“Delta” AY.99.1
“Delta” AY.34.2	“Delta” AY.34.1.1	“Delta” AY.88
“Delta” AY.86	“Delta” AY.105	“Delta” AY.106
“Delta” AY.126	“Delta” AY.90	“Delta” AY.20
“Delta” AY.80	“Delta” AY.85	“Delta” AY.90
“Delta” AY.46.6.1	“Delta” AY.56	“Delta” AY.88
“Omicron” BA.2	“Omicron” BA.1.1	“Delta” AY2.0
“Omicron” BA.1.1	“Delta” AY.55	“Delta” AY.39

## Data Availability

Information of sequence data are available in the Tables S1 and S2. All original and processed data for the results of the present study are available upon request.

## Supplementary Materials

The following supporting information can be downloaded at: https://www.mdpi.com/article/10.3390/v15102132/s1. Table S1: Accession numbers of Spike genes of five VOCs. Table S2: Information of ACE2 variants. Figure S1: Interaction profile of Spike protein and ACE2.
