# Supplementary material for: Emergence of Genomic Diversity in the Spike Protein of the “Omicron” Variant
Source: Viruses. 2023 Oct 21;15(10):2132. doi: 10.3390/v15102132 (PMC10612054; doi:10.3390/v15102132)
Supplement: Supplementary file 1 [file viruses-15-02132-s001.zip › Supplementary-Figure S1.pdf]

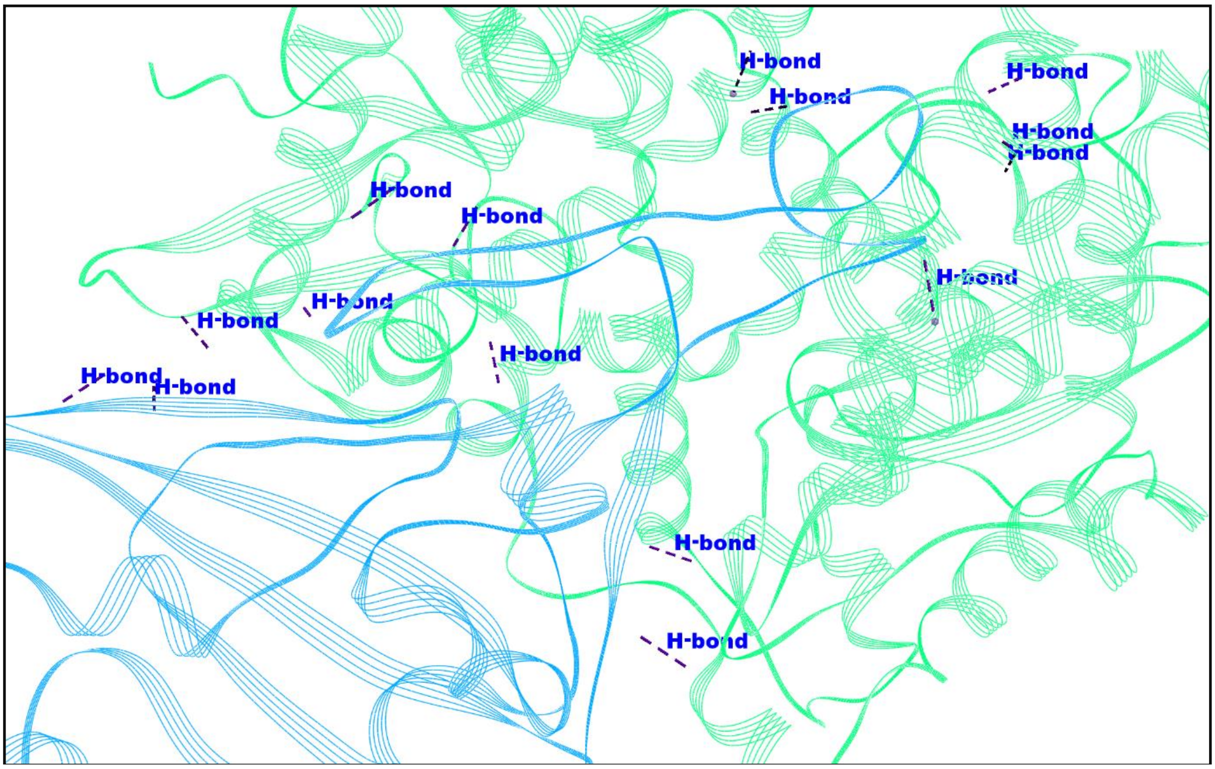
H-bond
H-bond
H-bond
H-bond
H-bond
H-bond
H-bond
H-bond
H-bond
H-bond
H-bond
H-bond
H-bond
H-bond
H-bond
H-bond

(a)

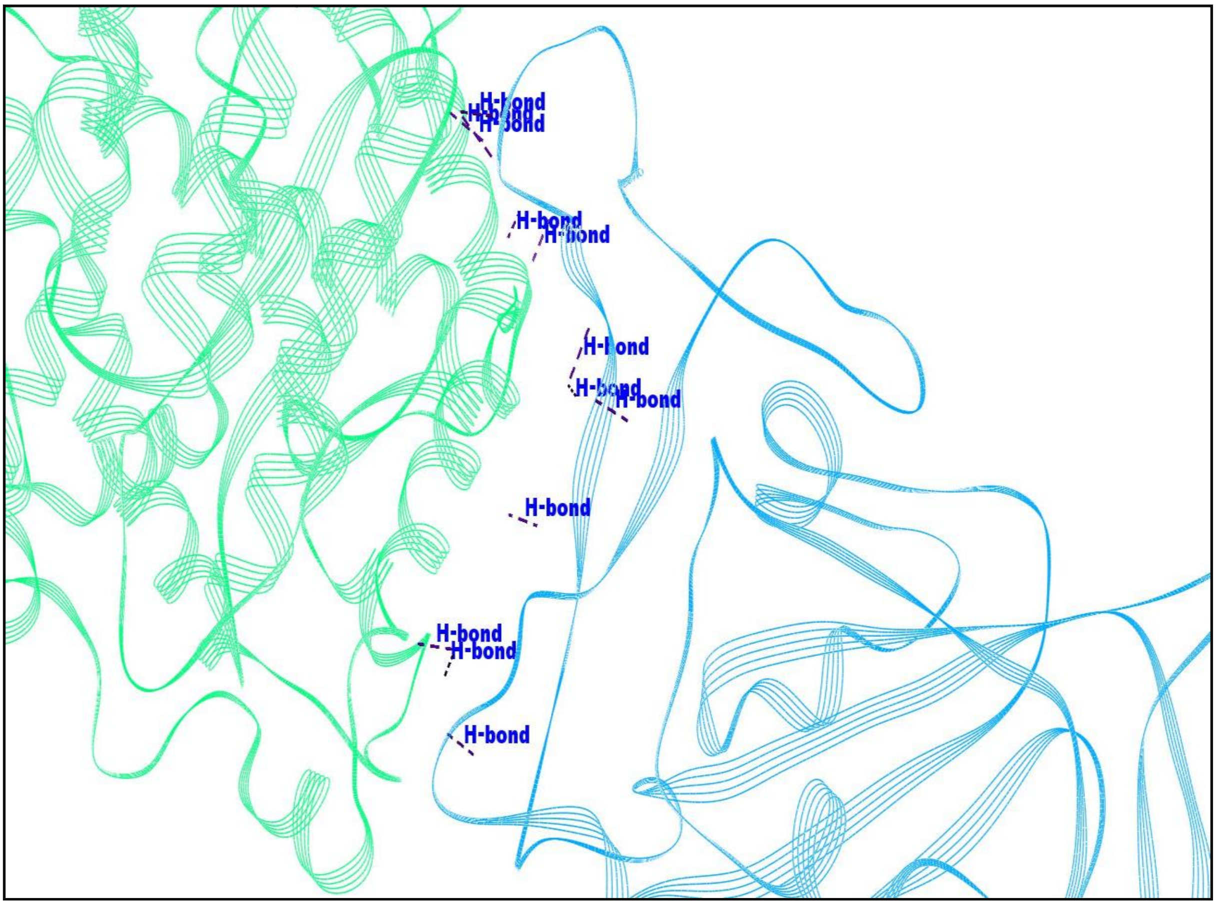
H-bond
H-bond
H-bond
H-bond
H-bond
H-bond
H-bond
H-bond
H-bond
H-bond

(b)

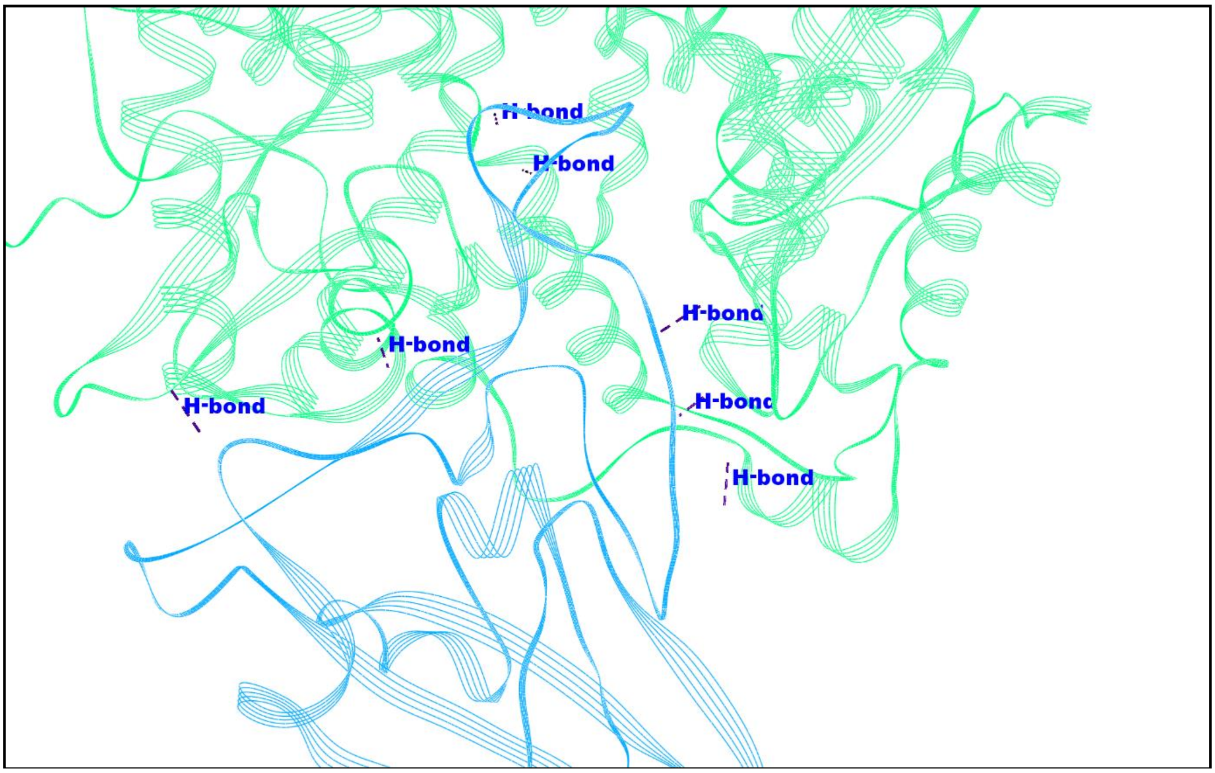
H-bond
H-bond
H-bond
H-bond
H-bond
H-bond
H-bond
(c)

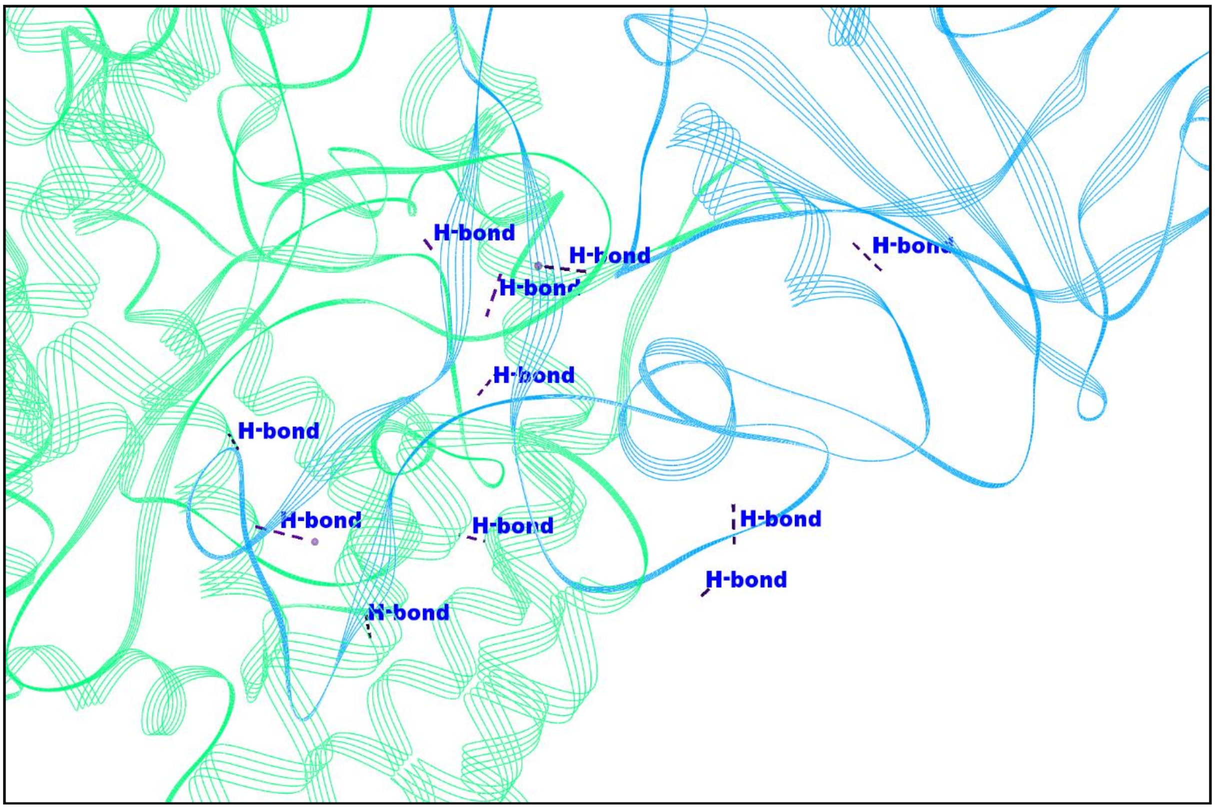
H-bond
H-bond
H-bond
H-bond
H-bond
H-bond
H-bond
H-bond
H-bond
H-bond
H-bond
(d)

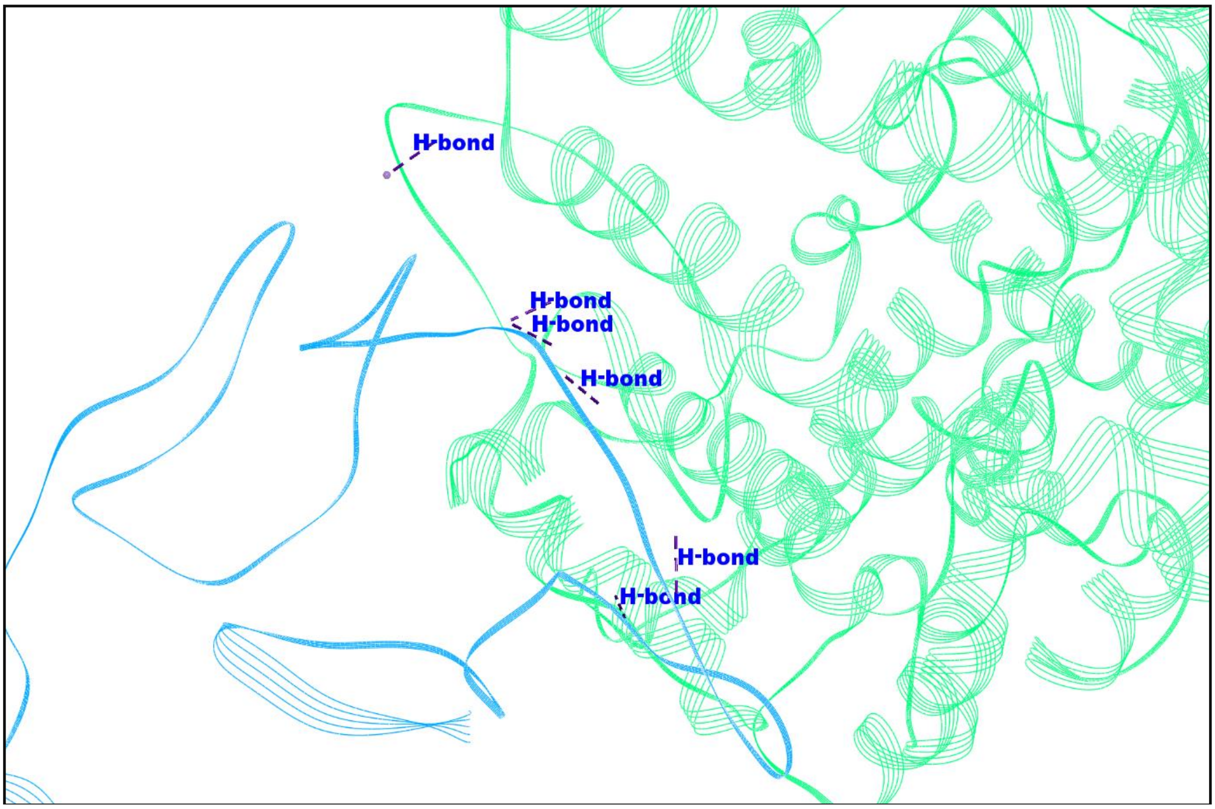

(e)

Figure S1. Visualization of H-bond interaction in the **(a)** spike (omicron)-ACE2 **(b)** spike (delta)-ACE2 **(c)** spike (gamma)-ACE2 **(d)** spike (beta)-ACE2 **(e)** spike (alpha)-ACE2 complex. Spike proteins are represented in cyan colour and ACE2 is represented in green colour. Hydrogen bonds (H-bond) are marked in blue. More hydrogen bonds in the spike (omicron)-ACE2 complex compared to other variants indicated highest stability of the spike (omicron)-ACE2 complex.
